# Genomic evidence for demographic fluctuations, genetic burdens and adaptive divergence in fourfinger threadfin *Eleutheronema rhadinum*

**DOI:** 10.1007/s42995-024-00276-4

**Published:** 2025-01-27

**Authors:** Jie Xiao, Wen-Xiong Wang

**Affiliations:** 1 School of Energy and Environment and State Key Laboratory of Marine Pollution, City University of Hong Kong, Kowloon, Hong Kong, China; 2 Research Centre for the Oceans and Human Health, City University of Hong Kong Shenzhen Research Institute, Shenzhen, 518057 China

**Keywords:** *Eleutheronema rhadinum*, Demographic history, Population divergence, Purifying selection

## Abstract

Declining populations and bottlenecks lead to the accumulation of deleterious mutations in fish populations. These processes also trigger genetic purging, which is a key genetic factor in reducing the deleterious burdens and increasing population viability. However, there is a lack of empirical evidence on the interaction between demographic history and the genome-wide pattern of deleterious variations. Here, we generated genome resequencing data of *Eleutheronema rhadinum* from China and Thailand, representing the major distribution of the species’ southern regions. *E. rhadinum* had exceptionally low genome-wide variability and experienced dramatic population expansions followed by continuous declines. The geographical divergence, which occurred ~ 23,000 years ago, shaped different demographic trajectories and generated different regional patterns of deleterious mutations in China and Thailand populations. Several lines of evidence revealed that this geographical pattern of deleterious mutation was driven by the purging of highly deleterious mutations. We showed that purifying selection had inbreeding-associated fitness costs and was more efficient against missense mutations in the Thailand population, which had the lowest genetic burden of homozygous deleterious mutations. Multiple evolutionarily conserved protein domains were disrupted by the loss-of-function mutations, posing a high probability of gene functionality elimination. Moreover, thermal and salinity genes (*Trpm3*, *Nek4*, *Gtf2f2*, *Cldn14*) were identified in genomic divergence regions of *E. rhadinum* among China and Thailand populations. Our findings highlight the importance of demographic history factors shaping the geographical patterns of deleterious mutations. The results serve to deepen our understanding of the adaptive evolution and divergence of *E. rhadinum* with implications for other marine fish.

**Supplementary Information:**

The online version contains supplementary material available at 10.1007/s42995-024-00276-4.

## Introduction

In Southern China, the fourfinger threadfin are traditionally considered to be the most precious fish, reflecting the cultural significance and high nutritional value attributed to the threadfin *Eleutheronema*, known for their delectable meat texture and flavor. The East Asian fourfinger threadfin, *Eleutheronema rhadinum*, belongs to a genus of fish that is mainly distributed in East Asia marine waters, including those of South and East China, Southeast Asia, and Japan (Motomura et al. 2002). To date, knowledge of the genetic study of *E. rhadinum* is still limited. The mitochondrial genome of *E. rhadinum* suggested its belonging to the family Polynemidae (Zhong et al. 2021) instead of the previously suggested Order *Mugiliformes* (Rosen 1964). Research conducted in China coastal waters and the Gulf of Thailand has revealed low nucleotide diversity, suggesting limited population structure in this species (Sun et al. 2013; Xiao et al. 2022, 2024). In recent decades, overexploitation and anthropogenic pressure may have caused a loss of diversity (Newman et al. 2011). However, the limited availability of genetic data currently impedes our understanding of its genomic diversity, evolutionary history, and subsequent assessment of long-term genetic viability and effective resource management for this species.

Escalating anthropogenic process has accelerated worldwide declines in species diversity (Jaureguiberry et al. 2022). These processes, which have implications for species management and conservation, lead to population structure changes, demographic shifts, and the emergence of genetic issues such as genetic drift, inbreeding depression, loss of genetic variability, and the accumulation of deleterious mutations (Charlesworth 2009; Frankham et al. 2014; Keller and Waller 2002). One of the effective approaches to mitigate the impacts of inbreeding depression and reduce the frequency of deleterious mutations is genetic purging (i.e., the process of increased pressure of natural selection against deleterious alleles prompted by inbreeding), which can minimize deleterious genetic loads (Glémin 2003). Studies have demonstrated the importance of purifying selection in reducing the number of deleterious mutations in endangered species characterized by small population sizes and low genetic diversity. For example, Robinson et al. (2018) found that purging of strongly deleterious mutations led to rapid recovery from bottlenecks in island foxes. Likewise, Grossen et al. (2020) presented genomic evidence of purifying selection of highly deleterious mutations in response to several bottlenecks in Alpine ibex. Demographic bottlenecks could result in an increase of inbreeding levels, leading to the accumulation of deleterious mutations in a population (Van Der Valk et al. 2019). Nevertheless, how demographic history and genome-wide patterns of deleterious variation interact with each other remains unknown in wild *E. rhadinum* populations.

The assessment of genomic data is a powerful means to investigate and understanding the genetic basis of local adaptation in non-model species, which is a central and fundamental goal of evolutionary biology (Tiffin and Ross-Ibarra 2014). However, the genetic adaptations of *E. rhadinum* to heterogeneous environments and the potential role of environmental selection in driving heterogeneous genomic divergence among populations remain poorly understood. Therefore, here we first generated genome-wide data from three populations found in Chinese waters (Zhanjiang, Jianghong, Zhoushan) and a fourth population found in waters of Thailand (Satun province), which was the first report of *E. rhadinum* in Thailand. Previous study showed that the *E. rhadinum* populations in China and Thailand showed local adaptations to different microhabitats (Xuan et al. 2022). To assess if differences existed, we investigated the genetic diversity, demographic history, local adaptation, and genetic divergence of *E. rhadinum* and then assessed the genetic viability of this species across different populations.

In our previous study, we reported the whole-genome resequencing data of its closely related sister species, *E. tetradactylum* (Xiao et al. 2023), which showed similar demographic history patterns among populations; we initially predicted that *E. rhadinum* would follow a similar pattern. However, in this study, we observed different demographic histories between the China and Thailand populations of *E. rhadinum*. To investigate the interaction between demographic history and the genome-wide pattern of deleterious mutations in *E. rhadinum*, we examined the genomic consequences of population decline and the impacts of recent demographic history changes on the patterns of deleterious mutations among *E. rhadinum* populations. Our study provided evidence of how changes in effective population size shaped the genetic burden in *E. rhadinum*. The results could provide valuable genomic resources and advance our understanding of the evolutionary history and mechanisms underlying the genetic purging and local adaptation of the fourfinger threadfin species.

## Materials and methods

### Sampling and sequence data processing

72 wild *E. rhadinum* individuals were collected from four populations throughout the China coastal waters and Thailand, including Zhanjiang (20.70°N, 110.55°E, Guangdong Province, China, *n* = 22), Jianghong (21.05°N, 109.45°E, Guangdong Province, China, *n* = 22), Zhoushan (22.51°N, 113.39°E, Zhejiang Province, China, *n* = 16) and Satun (6.291°N, 100.04°E,Thailand, *n* = 22). The DNA library of each individual was constructed with insert sizes of 300–500 bp and sequenced on the MGI-Seq 2000 sequencing platform (with an average depth of 11 ×) by Frasergen Biotechnology Co., Ltd (Wuhan, China). Quality assessment of the raw data was performed by FastQC (Andrews 2010), and then the clean data were generated by Trimmomatic (Bolger et al. 2014) after processing and removing the low-quality bases and artifact sequences. Clean data were mapped to the *E. rhadinum* reference genome using BWA (Li and Durbin 2010) with default parameters. Raw SNPs were called and then filtered by the GATK (McKenna et al. 2010) with the following parameters: QD < 2.0 || FS > 60.0 || MQ < 40.0 || MQRankSum < -12.5 || ReadPosRankSum < -8.0 || DP >  = 4. In addition, the SNPs with low allele frequency (MAF < 0.05) and low coverage (< 90%) at the population level were removed, and the filtered SNPs were used for further analysis. The filtered SNPs were then annotated using ANNOVAR (Wang et al. 2010) program, and all SNPs were classified into nonsynonymous, synonymous, UTR, exonic, intronic, intergenic, splicing, and non-coding RNA (ncRNA) of eight categories. Venn analysis implemented in R was used to detect the population or geography-specific SNPs. Difference in the variant numbers among populations was performed using the Mann–Whitney test implemented in R (https://CRAN.R-project.org/package=wmwpow).

### Population genetic analysis

Genetic diversity was measured by heterozygosity (*He*) and nucleotide diversity (*π*). *Tajima’s D* value was calculated for each individual and population. The genetic differentiation among populations was measured by Weir and Cockerham’s estimator of F_ST_. Specifically, π, F_ST_, and *Tajima’s D* were computed by Vcftools program with sliding windows of 100 kb with a step of 10 kb (Danecek et al. 2011). The expected *He* was calculated for nonoverlapping 1 Mb regions along the genome of each individual by Vcftools. The genome-level linkage disequilibrium (LD) decay of each population measured as parameter r^2^ was assessed using PopLDdecay (Zhang et al. 2019) with default parameters. Runs of homozygosity (ROH) per individual were estimated using PLINK (Purcell et al. 2007) with the following settings: -homozyg-density 50 -homozyg-window-kb 100 -homozyg-window-snp 50.

### Population structure and demographic history reconstruction

Neighbor-joining trees were constructed using TreeBest software (https://github.com/Ensembl/treebest) under the *p*-distances model with 1000 bootstrap replicates. The NJ with *p*-distances method offers a balance between accuracy and computational efficiency for phylogenetic tree construction based on whole genome-wide SNPs. The NJ tree was subjected to Figtree (https://github.com/rambaut/figtree/) for visualization and modification. Principal component analysis (PCA) was performed in GCTA (Yang et al. 2011). Population genetic structure and ancestry proportions were determined using the ADMIXTURE program (Alexander et al. 2009) with the number of genetic clusters (K) from K = 1 to K = 10.

The pairwise sequentially Markovian coalescent (PSMC, https://github.com/lh3/psmc) model was used to reconstruct the demographic histories for each population with the following parameters: -N 30 -t 15 -r 5 -p 4 + 25*2 + 4 + 6. We also conducted multigenome analysis for demographic history inference using another SMC +  + (Terhorst et al. 2017). We set the generation time (g) of *E. rhadinum* as 4.5 years, and the estimated nucleotide mutation rate (μ) as 1.9 × 10^–8^ mutations per site per generation to convert the resulting scaled values into years and individuals (Horne et al. 2012). We conducted the SMC +  + analyses based on the “estimate” and “split” method with composite likelihood for demographic inference and split models.

### Inbreeding pattern and deleterious mutation loads

Population inbreeding level measured by genomic inbreeding coefficients (F_ROH_) and based on the ROH was computed by the PLINK program. SnpEff (Cingolani et al. 2012) was used to identify the loss-of-function (LoF, mutations with the gain of a stop codon or splice-site-disrupting single nucleotide mutations), missense, and synonymous variations in coding regions based on the filtered SNP calls. The deleterious missense variants were filtered among missense mutations using the SIFT4G program (Vaser et al. 2016). As an indication of genetic load, we calculated the ratio of the number of homozygous LoF or deleterious missense mutations divided by the number of synonymous mutations (Fay et al. 2001). The protein sequence of LoF-affected genes was annotated using InterProScan v.5.33 (Jones et al. 2014) to identify the conserved protein family (PFAM) domains. GO and KEGG functional enrichment analyses of candidate genes affected by homozygous LoF were performed to determine the potential functions using TopGO (Alexa and Rahnenfuhrer 2010) and KOBAS (Xie et al. 2011).

### Identification of genomic regions under selection

Genetic differentiation (F_ST_) and nucleotide diversity (*θπ* ratio) indexes were combined to detect the candidate selected region for each population (Wu et al. 2014). We calculated the F_ST_ and *θπ* of each population using sliding windows of 100 kb with a step of 10 kb along the genome, and the windows with the top 5% of values for the F_ST_ and log2(*θπ* ratio) simultaneously served as the candidate outliers under selection. The corresponding SNPs and genes of the candidate selected region were identified and then subjected to KEGG and GO enrichment analysis using TopGO (Alexa and Rahnenfuhrer 2010) and KOBAS (Xie et al. 2011).

## Results and discussion

### Genome-wide variations of *E. rhadinum*

**Fig. 1:**
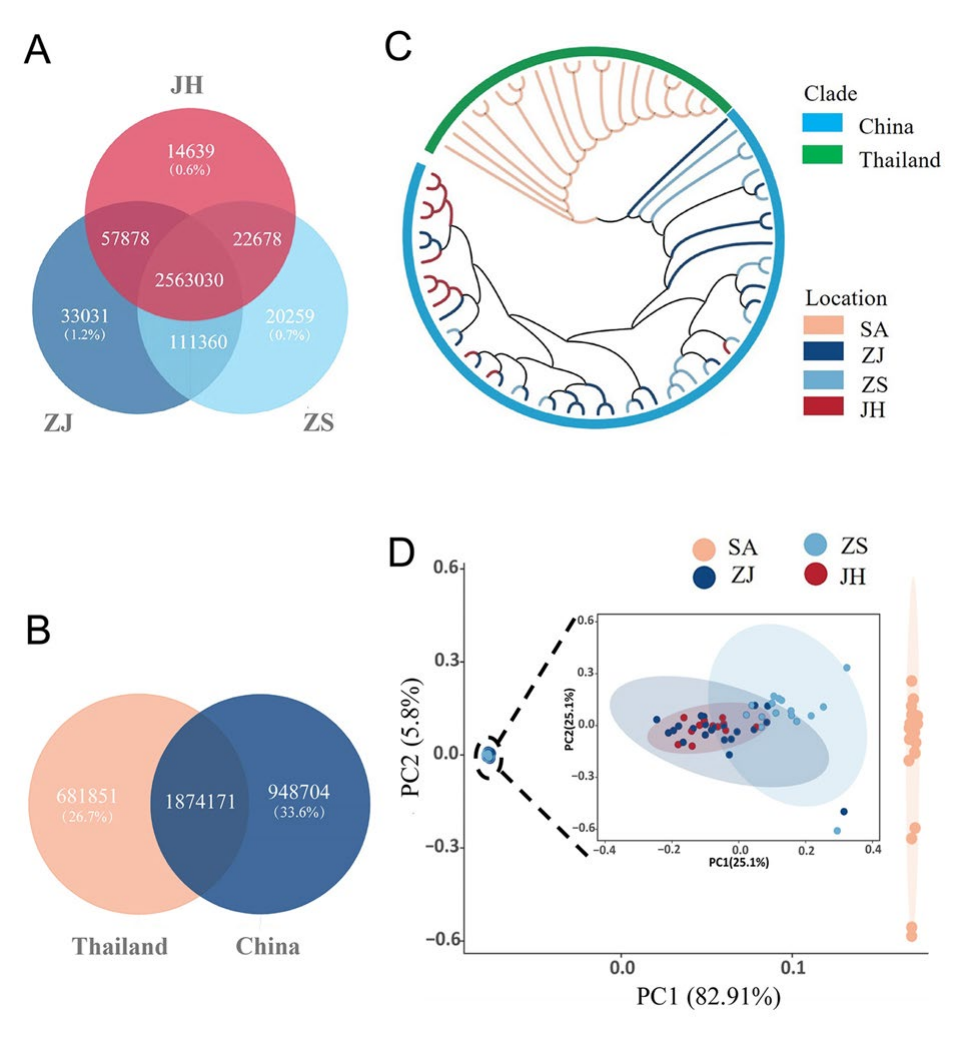
Genome-wide variations and phylogenetic relationships of *E. rhadinum*. **A** Venn diagrams for shared variants detected in China populations. **B** Venn diagrams for variants shared between China and Thailand populations. **C** Phylogenetic tree of four fish populations based on whole-genome SNPs. **D** PCA plot of four fish populations

We collected 72 wild *E. rhadinum* individuals from China and Thailand. The China populations were collected from Jianghong (JH, *n* = 12), Zhanjiang (ZJ, *n* = 22), and Zhoushan (ZS, *n* = 16), whereas the Thailand population was collected from Satun Province (SA, *n* = 22), which covers the main distribution of southern regions of wild *E. rhadinum*. We generated ~ 516 Gb of data for the 72 samples, with an average sequencing depth of ~ 11 × for each individual. The clean data were mapped to the *E. rhadinum* genome (accession number: PRJNA1043948), resulting in an average ~ 99.5% alignment rate and ~ 98.7% genome coverage (Supplementary Table S1). After detecting and filtering raw SNPs, we identified ~ 3.5 million SNPs in the *E. rhadinum* genome, and the average number of SNPs for each individual was ~ 2.3 million (Supplementary Table S1). The number of SNPs per population was significantly different (Mann–Whitney test, *p* < 0.001), with the SA population having approximately 1.5 times more SNPs than the three China populations (Supplementary Fig. S1). We found ~ 2.56 million SNPs (99%) that were shared among three China populations (Fig. 1A), whereas ~ 1.8 million SNPs were shared between China and SA populations (Fig. 1B). Moreover, we identified ~ 0.6 million (Satun) and ~ 0.9 million (China) regional-specific SNPs (Fig. 1B), which might have potential contributions to local adaption. Among all SNPs, ~ 55% were located in or near genes (Supplementary Table S1), including open reading frame (Upstream, 16.25%; downstream, 15.8%), introns (33.23%) and untranslated regions (UTRs, 2.4%). Furthermore, 170,731 SNPs were located in protein-coding exons, resulting in 48,143 nonsynonymous mutations, with 646 stop codon changes potentially influencing the function of corresponding genes.

### Phylogenetic relationships and population structure of *E. rhadinum* populations

**Fig. 2:**
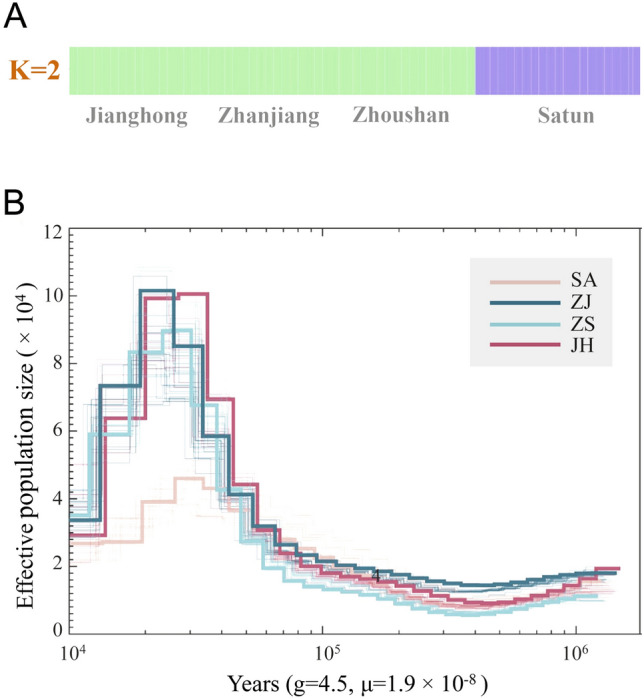
Population structure and demographic history of *E. rhadinum*. **A** Population structure analysis of *E. rhadinum* by ADMIXTURE. CV errors are low when K = 2 to 4 (see Supplementary Fig. S3). Four populations are clustered from each other when K = 4. **B** Demographic histories of the four populations based on the PSMC model. The time axis is logarithmically transformed

To determine the phylogenetic relationships among the four populations, we constructed a phylogenetic neighbor-joining (NJ) tree based on the genome-wide SNPs (Fig. 1C), revealing that the three China populations and the SA population could be separated into two different clusters. Principal component analysis (PCA) and admixture analysis supported the population structure as evidenced in the NJ tree (Fig. 1D). The PCA plot of the 72 individuals showed a separation between the China populations and the SA population, which could be connected to geographic barriers (Xiao et al. 2022). However, the three China populations could not be distinguished from each other, consistent with the phylogenetic results. Results from the admixture analysis also provided evidence of clustering that the SA population formed a separate group when K = 2 (Fig. 2A; Supplementary Figs. S2, S3). The present results revealed a geographic structure signal between the three China populations and the SA population from Thailand, suggesting potential population subdivision or isolation between the SA and China regions.

### Demographic and divergence history of *E. rhadinum*

Reconstruction of demographic history provides clues to understand how the pattern of genetic diversity is shaped in populations, with evolutionary implications for assessing the adaptive potential. Our demographic inference showed that *E. rhadinum* experienced population expansion followed by a decline, with the peak ancestral effective population size occurring ~ 30,000 years ago (Fig. 2B; Supplementary Fig. S4). Additionally, we observed different demographic trajectories between the three China populations and the SA population, although both experienced population expansion and bottlenecks. The size of the population expansion was different, and the effective population size of three China populations was ~ two times of the SA population, implying that the three China populations during the Last Glacial Maximum period had a larger population size than the Satun *E. rhadinum* from Thailand. We also observed a split of the effective population size curves between the China and SA populations, which occurred ~ 23,000 years ago based on the SMC +  + inferences (Supplementary Fig. S4). This was possibly the initial geographic divergence time of the China and SA populations, and most likely caused by the last glaciations or geographical isolation. These data suggested that the China and SA *E. rhadinum* populations shared the same ancestor and that the lack of separation of the China populations might be due to intensive population migration and exchange. It is noteworthy that historical patterns of population bottleneck have also been observed in many marine fish, such as *Sebastes schlegelii, S. koreanus*, *S. nudus*, and *Syngnathus typhle* (Knutsen et al. 2022; Xu et al. 2019). These concordant patterns suggested that the evolutionary history of most marine fish has been influenced by global glaciation as well as extreme cold climates.

In accordance with the previous mtDNA and morphometric results of *E. rhadinum* (Xiao et al. 2022), considerable genetic divergence was also observed between the China and SA populations (Fig. 2A). Notably, such a divergence occurring during the last glaciation period might reflect the historical isolation as the geological events might create a vicariant barrier between the South China Sea and Thailand (Liao et al. 2007). One of the key events was the global drop in sea levels due to the extensive glaciation of large landmasses, which might have caused the emergence of land bridges or the alteration of existing landforms, creating a physical barrier between the two regions and resulting in the split between the *E. rhadinum* populations from China and Satun (Wurster et al. 2010). The different demographic histories and population sizes between China and Satun populations might have resulted from geographic or climatic differences. Moreover, the demographic history revealed that the effective population size of *E. rhadinum* had gradually decreased through time, which could explain the extremely low levels of genetic diversity in *E. rhadinum* populations.

### Genome-wide diversity and differentiation of *E. rhadinum*

**Fig. 3:**
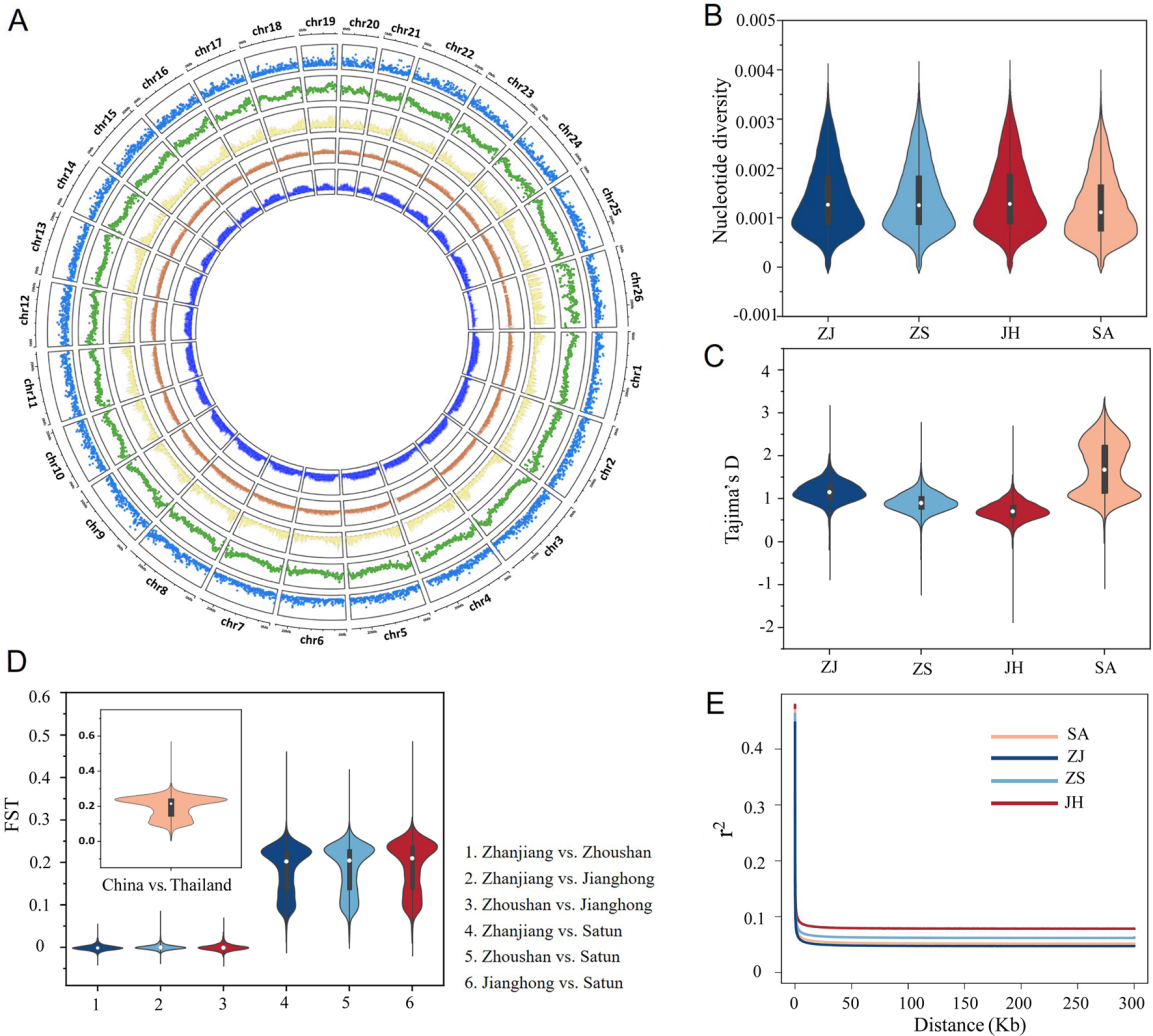
Genomic features and genetic diversity of wild *E. rhadinum* populations. **A** Diagram of genome-wide variants and genetic characteristics. Circles from outermost to innermost indicated, respectively, 26 chromosomes (chr. 1–26), gene density, SNP density, nucleotide diversity, *Tajima’D* value, and F_ST_ value between China and Thailand populations (Supplementary Table S2). **B** Nucleotide diversity over 100-kb nonoverlapping windows. **C** Average genome-wide *Tajima’s D* value. **D** Average estimates of genetic differentiation (F_ST_) between pairwise populations. **E** The decay of LD measured by r^2^ for the four populations

The genome-wide nucleotide diversity was calculated to estimate the genetic diversity of different *E. rhadinum* populations (Fig. 3A). We observed that the three China populations had relatively higher levels of genetic diversity than the SA population (Fig. 3B). Among marine fish species for which genome-wide variations were available, *E. rhadinum* (π = 1.24 ~ 1.4 × 10^–3^) had lower genetic diversity than *Oncorhynchus mykiss* (π = 2.3 × 10^–3^), *Sillago japonica* (π = 2.2 × 10^–2^), *Clupea harengus* (π = 3 × 10^–3^) (Barrio et al. 2016; Gao et al. 2018; Han et al. 2021), and even the closely related sister species *E. tetradactylum* (π = 1.71 × 10^–3^). In addition, *Tajima’s D* values for the Thailand population were higher compared to the China populations (Fig. 3C), which could be explained by the lower rare allele frequencies. The indicator of population differentiation among the *E. rhadinum* populations suggested that the geographic divergence F_ST_ was around 0.2, while no obvious differentiation was observed among the sympatric population (Fig. 3D). The linkage disequilibrium (LD) decay reflects natural selection on the genome of a population. Then, we estimated the LD for different *E. rhadinum* populations. The overall LD decay distance with decay to half of its maximum value in *E. rhadinum* was 10 kb (Fig. 3E), which was similar to its sister species *E. tetradactylum* (Xiao et al. 2023). The LD decay speed varied among different groups, with the slowest LD attenuation in the JH population, implying different groups experience different selection pressures. Overall, these data revealed a low level of genetic diversity in *E. rhadinum*, indicating their decreased adaptive potential in changing environments, and may thus require conservation actions.

### Genetic purging of deleterious burden in *E. rhadinum*

**Fig. 4:**
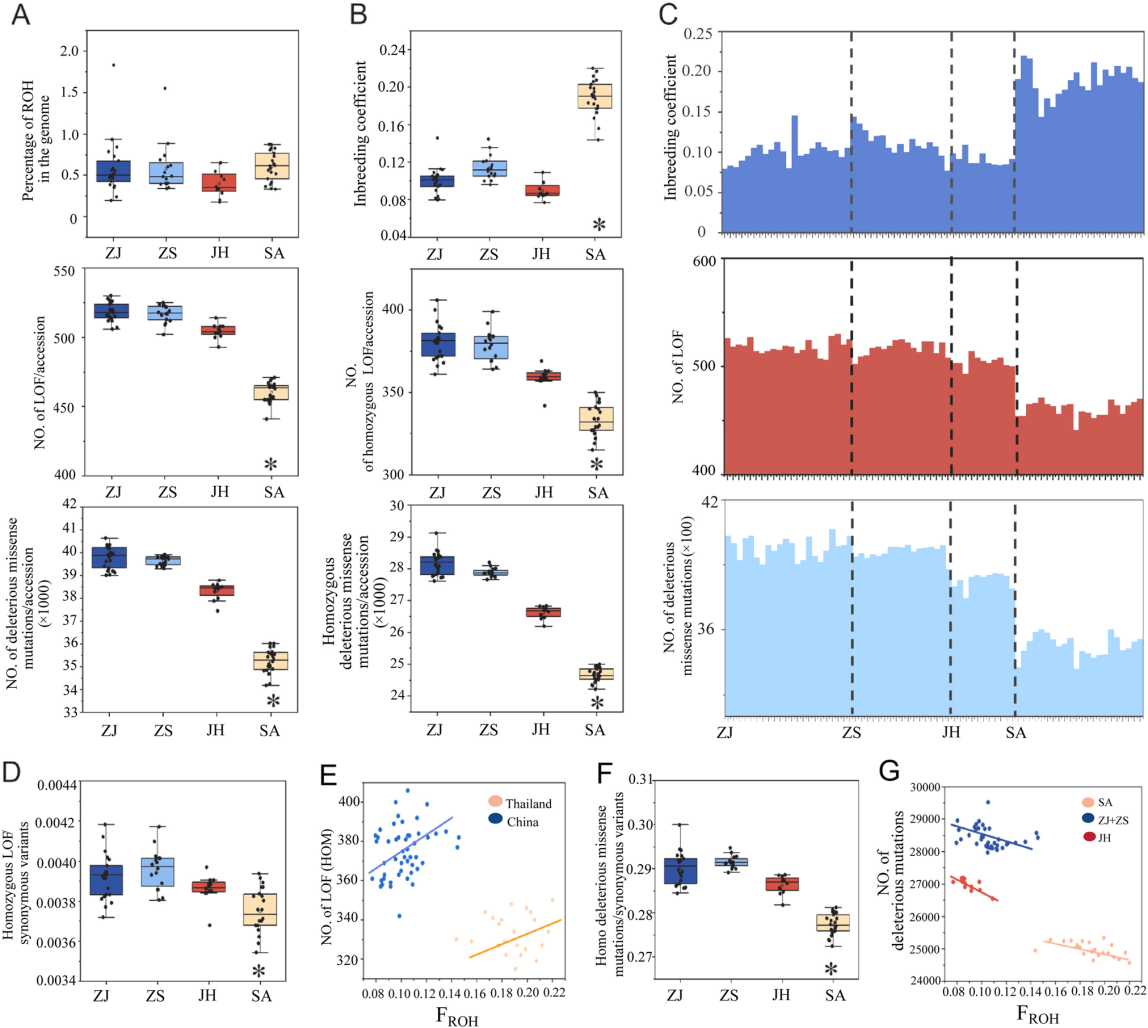
The genetic burden of LoF and deleterious missense mutations and purging of deleterious mutations in *E. rhadinum*. **A** Individual inbreeding coefficients estimated using ROH. **B** Percentages of ROH on genome. **C** Top: inbreeding level of each sample measured by ROH; middle: from left to right showed the average number of LOF, the homozygous LOF mutations of each population, and the number of LoF mutations of each sample; bottom: from left to right showed the average number of deleterious missense mutations, the homozygous deleterious missense mutations of each population, and the number of deleterious missense mutations of each sample; Ratio of derived homozygous **D** LoF and **E** deleterious missense variants to synonymous variants per individual. Relationships between inbreeding of homozygous **F** LoF mutations and **G** deleterious mutations (LoF + missense mutations)

Considering the continuous and gradual decrease in the population size of *E. rhadinum*, we determined the genomic impacts of population decline on *E. rhadinum* by identifying the genetic signatures of inbreeding and deleterious mutations. We examined the heterozygosity and runs of homozygosity (ROH) to determine the genetic impacts of continuous population decline on *E. rhadinum*. *E. rhadinum* showed low genome-wide heterozygosity (0.23 in three China populations, and 0.205 in SA) relative to *E. tetradactylum* (0.25) (Supplementary Fig. S5), whereby a low level of nucleotide diversity indicated their decreased adaptive potential in the natural environment (Xiao et al. 2023). We detected stretches of short (100 Kb up to 500 Kb) along with a few long (1 Mb up to 10 Mb) ROH in *E. rhadinum*, which accounted for around 0.45–0.55% of the genome (Fig. 4A; Supplementary Table S3). Short ROHs were more prevalent throughout the *E. rhadinum* genome. The SA population had the largest fraction of ROH in the genome and lower heterozygosity levels in comparison to the three China populations, which was likely explained by the small effective population size as indicated by demographic history of SA *E. rhadinum*. Short ROHs tended to be formed by common ancestry in the distant past (Ceballos et al. 2018) and were common in populations that experienced a bottleneck. The large amount of short ROHs as well as low heterozygosity in populations could be explained by the continuous decrease in population size, particularly in the SA population from Thailand, consistent with its demographic history.

We quantified the population inbreeding using ROH and observed a relatively high level of inbreeding in the SA population (*P* < 0.05, Fig. 4B), which could be explained by the ancestral inheritance or potential ancient bottleneck in the SA population. In our study, we observed an inverse relationship between the inbreeding level (F_ROH_) and the effective population size, similar result also has been reported in previous study (Khan et al. 2021). Specifically, we found that the Thailand population, which experienced a small population size over an extended period, exhibited a higher level of inbreeding. Next, we investigated the prevalence of deleterious mutations, as it is closely related to the population inbreeding and bottlenecks. Overall, *E. rhadinum* carried a number of deleterious variants (LoF and deleterious missense variants), which could be explained by the recent bottleneck and continuous population decline (Fig. 4C). The three China populations harbored comparable numbers of deleterious mutations, with an average of 1.25-fold increase in deleterious variants, and a 1.5-fold decrease in inbreeding level relative to the SA population, implying a substantial genetic load (Fig. 4C).

Population declines lead to the accumulation of deleterious mutations in populations, which builds up a genetic burden that threatens their survival (Lynch et al. 1995). Nevertheless, this process could also trigger genetic purging in small populations, thus reducing the deleterious burden and increasing population viability. Demographic analysis showed that the SA population was small and isolated relative to the three China populations, which were relatively large and connected. The three China populations carried the least inbred and the largest mutation loads of LoF and missense mutations (Fig. 4D, E). Alternatively, the small SA population had a much higher average population inbreeding level but lower mutation loads, and one of possible reasons could be ascribed to genetic purging or genetic drift in SA population in light of our theoretical predictions.

Next, we evaluated the relationship between inbreeding and deleterious mutations among populations. We only calculated the number of homozygous deleterious mutations because most of them were partially recessive, and thus the homozygous mutations were more likely informative of the fitness cost of individual inbreeding (Khan et al. 2021). We observed that the number of homozygous deleterious mutations was larger than that of heterozygous mutations, which could reflect the main contribution of the homozygous mutations to the genetic burden (Supplementary Fig. S6). The number of homozygous LoF mutations was proportional to the inbreeding level in all populations, indicating the cost of genetic fitness along with increased population inbreeding (Fig. 4F). However, we observed a higher population inbreeding associated with lower deleterious mutations (Fig. 4G), consistent with a previous study that suggests purifying selection is more efficient against missense mutations in small and isolated population (Khan et al. 2021). We compared the mutation loads and inbreeding in *E. rhadinum* with its closely related sister species *E. tetradactylum* (Xiao et al. 2023). There was a lower load of deleterious mutation and higher inbreeding level in *E. rhadinum*, possibly explained by the genetic purging of deleterious burden that removed part of putatively deleterious mutations in *E. rhadinum* due to the generally small population size (Supplementary Fig. S7A–D). Together, our study suggests that the SA population with the smallest historical Ne has the lowest genetic burden of homozygous deleterious mutations compared to the relatively large and connective China populations.

Overall, our present study reveals the signatures of purging for putatively deleterious mutation in *E. rhadinum*. The general decrease in deleterious mutation represents a feature involved in the long-term small population size (Xue et al. 2015). However, this population shows a relatively higher inbreeding level, indicating a potential fitness cost of purging of some deleterious mutations. Given the increasing inbreeding level, conservation management of small *E. rhadinum* population still needs to be addressed to aid the long-term persistence of populations (Willi et al. 2022).

### Impact of deleterious LoF mutations in *E. rhadinum*

**Fig. 5:**
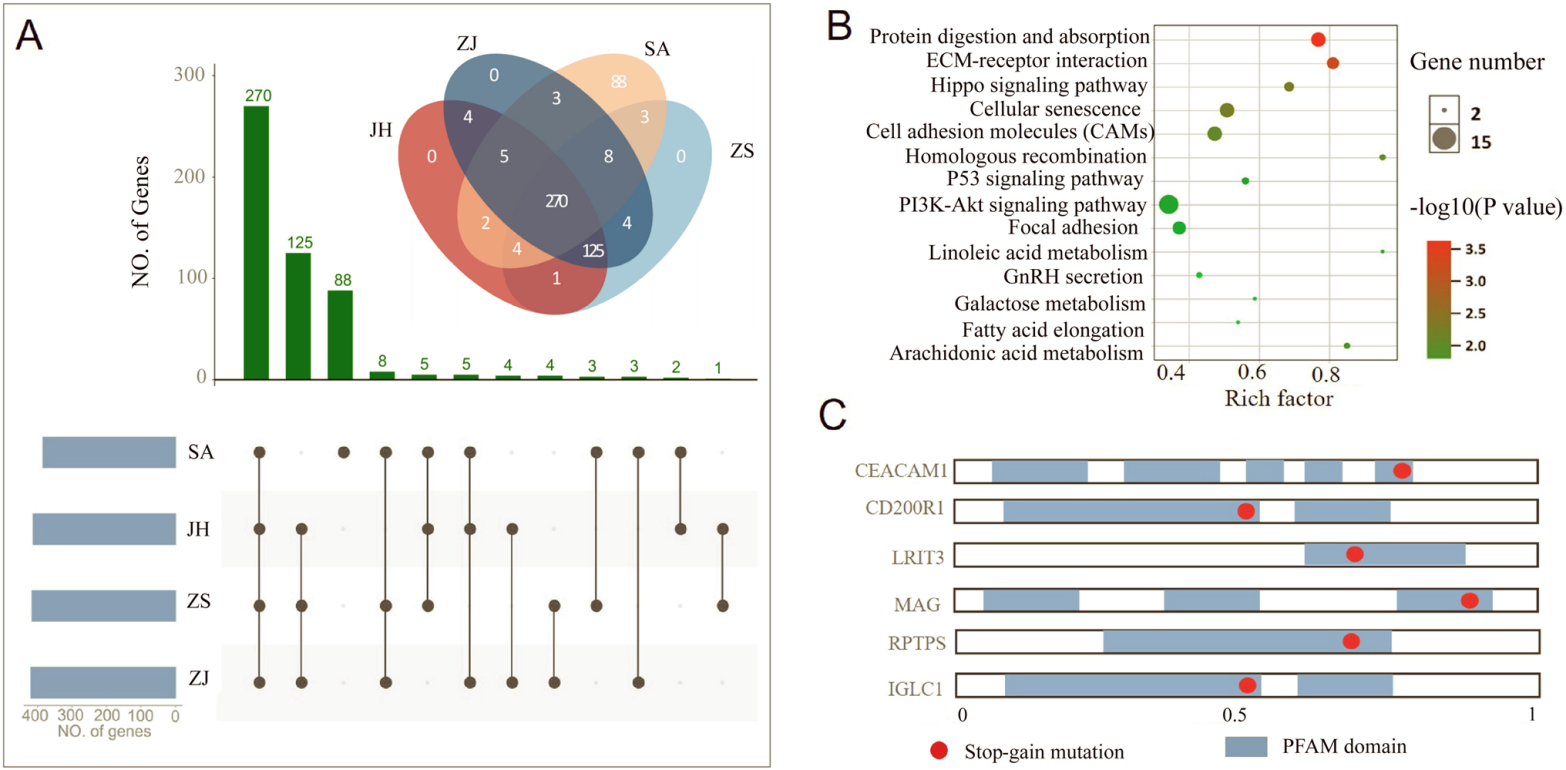
Impact of deleterious LoF mutations in *E. rhadinum*. **A** Distribution of the number of homozygous LoF genes in four populations. **B** KEGG enrichment analysis for LoF genes. **C** The localization of protein family domains (PF13855) is highlighted in blue. Red dots represent the relative position of the LoF mutation in protein-coding sequences of *E. rhadinum*

517 genes containing homozygous LoF mutations were identified in *E. rhadinum* (Fig. 5A). Most LoF genes were shared among populations, which account for ~ 52% of total LoF genes. About 25% and 17% of private LoF genes were discovered in the three China populations and the SA population, respectively, implying that the patterns of deleterious mutations were more likely to evolve in similar geographic environments. We speculated that the geographical differentiation between the China and SA *E. rhadinum* might generate such regional differences in the patterns of deleterious mutations and relevant genes. These LoF genes were significantly enriched in genes related to protein digestion and absorption, ECM–receptor interaction, cellular senescence, cell adhesion molecules, and PI3K–Akt signaling pathway (Fig. 5B). These LoF mutations are located in genes primarily responsible for the immune system and environmental adaptation. This may increase their susceptibility to diseases and pose a potential threat to their survival in the wild. We then analyzed the impact of LoF mutations by predicting the protein truncation. Most genes with conserved protein family (PFAM) domains were disrupted by LoF mutations (Supplementary Table S6). Among them, the genes with immunoglobulin domains, closely related to the immune system, were largely affected by the mutation, which had a high potential to stop their functional protein coding (Fig. 5C). We speculated that the LoF mutation via disrupting the conserved domain posed a high probability of impacting fitness by reducing or eliminating gene functionality. However, the realized fitness impacts of LOF mutation were largely unknown, while most were expected to be deleterious; thus, studies need to further assess the functionally of the effect of these mutations in fish. These results provided genomic evidence that the evolutionarily conserved protein domains were disrupted by the LoF mutations.

### Genetic basis of ecotype divergence and adaptive evolution in *E. rhadinum*

**Fig. 6:**
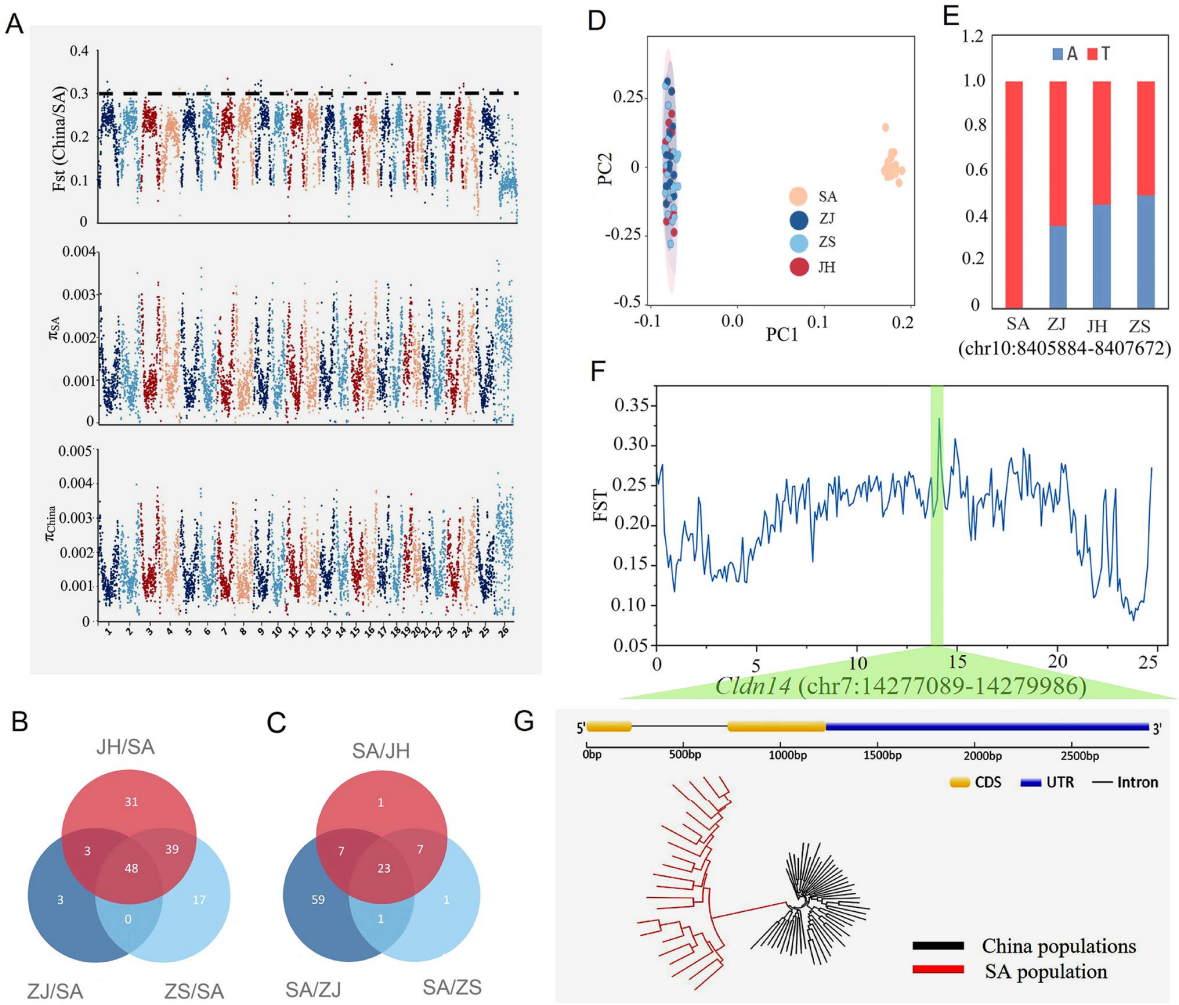
Genomic divergence and adaptive evolution in *E. rhadinum*. **A** Manhattan plots of F_ST_ and π between and within *E. rhadinum* populations using a 100-kb nonoverlapping window. Horizontal dotted lines represent the genomic regions of high divergence in each chromosome. Overlapping candidate genes under sweep selective in SA population **B** and three China populations **C** based on the Venn diagram. **D** PCA of thermal adaptations genes. **E** Allele frequency of one SNP within thermal adaptation gene *Trpm3* between China and SA populations; red and blue represent two types of bases at this locus. **F** Genomic differentiation (F_ST_) between SA and China population for chromosome 7. The highly divergent genomic region was labeled using a dashed vertical line. **G** Characterization of a salinity-responsive gene *Cldn14* in *E. rhadinum*, which was under a highly diverged region between SA and China populations. The phylogeny of the gene *Cldn14* showed a clear separation of SA and China populations

In addition to morphological differentiation among populations, the China and Thailand populations also showed local adaptations to different microhabitats (Xuan et al. 2022). To explore the underlying genomic drivers of ecological adaptation and divergence, we compared the genomes between the China and SA populations to identify the regions under selection. The selective sweep regions were scanned using a combination of F_ST_ and pairwise nucleotide diversity (θπ_China/SA_) (Fig. 6A). We identified 140 genes under putative selection regions as potential genes associated with local adaptation in Satun from Thailand (Fig. 6B). Functional enrichment analysis suggested that these candidate genes were significantly enriched in pathways related to digestive system, energy metabolism, endocrine and immune system, and environmental adaptation, potentially contributing to ecological adaptation (Supplementary Tables S7–S8).

Water temperature is one of the most important abiotic factors influencing the phenotypes and habitats of aquatic organisms (Grummer et al. 2019). Satun Province, located on the Andaman coast of Southern Thailand, features economically important fishery resources of the Andaman Sea, with an average water temperature of 30 °C and water salinity of 30 (Samphan 2016). Mechanistically, fish on the Andaman coast of Southern Thailand were frequently exposed to higher water temperatures relative to China coastal waters; thus, these candidate genes under selected genomic regions might be associated with thermal adaptation in Thailand. Notably, the PCA results based on SNPs of these candidate genes related to thermal adaptations in the Thailand population also divided all these individuals into two clades (Fig. 6D), supporting the contributions of these candidate genes to the genetic divergence. Among these genes, for example, the *Trpm3* gene, considered to be a thermoreceptor, was involved in temperature sensing and played an essential role in the heat stress of *Manila clams* (Massullo et al. 2006; Zhang et al. 2023). For the Thailand population, the *Trpm3* gene showed clear differentiation from those in China populations (Supplementary Fig. S8), and the allele frequencies of the SNPs within the gene *Trpm3* showed high values in the Thailand population than China population (Fig. 6E), which might facilitate the adaptation of fish to the high-temperature environment. Moreover, multiple genes (serine/threonine-protein kinase Nek4 (*Nek4*), cathepsin B (*CathB*), calreticulin (*CRT*, *Calpain2*) (Akbarzadeh et al. 2018; Liu et al. 2019, 2023; Visudtiphole et al. 2010) that were closely involved in thermal stress were also located within these regions (Supplementary Table S8). Thus, multiple genes in the genomic regions of the Thailand population under selective sweeps associated with thermal stress played essential roles in the ecological adaptation of *E. rhadinum*.

Additionally, a 0.3 Mb block from 14.1 to 14.4 Mb on Chromosome 7 showed significant differentiation between the three combined China populations and the SA population, which had the highest peak value (Fig. 6F). This region harbored several protein-coding genes (e.g., *Epha6*, *Arf6, Fbxo47*, *Cldn14*, *Ranbp2*, *Gtf2f2*) with various biological functions related to reproduction, growth, immune system, and environment adaptation. A gene encoding claudin *Cldn14* resided within this region, which pleiotropically contributed to the osmoregulatory physiology of the epidermis and might be responsive to salinity changes in the integument of fish (Bui and Kelly 2014; Bui et al. 2010). A study showed that claudin proteins contributed to seawater acclimation in the euryhaline pufferfish *Tetraodon nigroviridis* (Bui and Kelly 2014). The phylogeny of *Cldn14* clearly showed that the SA population was separated from the three China populations (Fig. 6G). In addition, previous study revealed that *Gtf2f2* (general transcription factor IIF 2) involved in osmoregulation played important roles in response to salinity in European whitefish (Papakostas et al. 2012), which was also discovered in highly differentiated regions between China and SA population. The results suggest that the thermal and salinity adaptation genes are under potential genomic divergence region among different *E. rhadinum* populations and may play roles in fish adaptation of high temperature and salinity in Thailand water environment compared to fish in China coastal water. Overall, our study, therefore, suggests that thermal and salinity-related genes identified between the China and SA populations might contribute to the ecological divergence and adaptation in *E. rhadinum*.

Geographically distinct populations exposed to similar environmental conditions evolve similar genotypic and phenotypic traits. Some shared genomic regions among three China populations were highly diverged from the SA population in Thailand, suggesting that these genomic regions may underlie shared targets of selection in wild evolution under changeable water environment in the South China Sea. Considering the less geographic isolation and similar temperature environments, the shared genomic regions with 99 selection genes among three China populations could be considered as evidence for ecological adaptation of *E. rhadinum* to China coastal waters (Fig. 6C). Functional enrichment analysis revealed that most of these genes were enriched in retrograde endocannabinoid signaling, GABAergic synapse, and neuroactive ligand–receptor interaction pathways and multiple ion transporter GO terms (Supplementary Tables S9–S10), which are closely involved in environmental stress response in fish (Facciolo et al. 2010; Feng et al. 2022; Zhang et al. 2017). Moreover, some genes (e.g., *Mhc7*, *Map3k14*) involved in cold stress were also detected in three China populations (Akbarzadeh et al. 2018; Velmurugan et al. 2019). Hence, our study provides evidence of adaptive evolution in genes related to cold stress which are functionally necessary for the environmental adaptations of *E. rhadinum* in China coastal water. Moreover, similar genetic evolution patterns induced by temperature have been also reported in *Sillago japonica* and *Gadus morhua* (Bradbury et al. 2010; Han et al. 2021). Overall, our study, therefore, provides novel insights into the genetic mechanism of ecological adaptation in wild *E. rhadinum*, with implications for other fish species in the China coastal waters.

## Conclusion

The fourfinger threadfin *E. rhadinum* is well known for its high nutritional value in China; however, its genomic diversity and evolutionary history are relatively obscure. Here, we comprehensively investigated the genetic diversity, phylogenetic relationship, demographical history, and genomic consequences of population decline in *E. rhadinum*. We observed that the genetic diversity of *E. rhadinum* was substantially lower compared to that of other wild marine fish species, emphasizing the vulnerability of its populations. The geographic divergence between the China and Thailand populations, estimated to have occurred around 23,000 years ago during the last glaciation period, has shaped different demographic trajectories. The Thailand SA population, which was isolated from the three large and connective China populations, showed the highest inbreeding level, but lowest genetic burden of homozygous deleterious mutations, suggesting the potential impact of genetic purging. Furthermore, our study identified genes responsible for the immune system and environmental adaptation that were disrupted by loss-of-function (LoF) mutations. These LoF mutations, particularly in genes with conserved protein family domains, posed a potential impact on relevant genes by eliminating their functionality. Multiple thermal and salinity-related genes identified in genomic divergence regions between the China and SA populations might contribute to the ecological divergence and adaptation in *E. rhadinum*, whereas the three geographically distinct China populations showing similar evolutionary patterns were likely induced by similar environmental conditions in China coastal waters. Overall, our study provides the first genome-wide genetic data for this cryptic and valuable fourfinger threadfin species. These findings emphasize the importance of implementing conservation initiatives aimed at improving the management of regional and uniquely adapted populations in *E. rhadinum*. By considering the genetic diversity, demographic history, and genomic consequences of population decline, we can develop targeted conservation strategies that address the specific needs of *E. rhadinum* populations in different regions. This will contribute to the long-term preservation of this species and the maintenance of its ecological and economic value.

## Supplementary Information

Below is the link to the electronic supplementary material.

Supplementary file1 (PDF 521 KB)

Supplementary file2 (XLSX 943 KB)

## Data Availability

The sequencing data generated in this study have been submitted to the NCBI BioProject database (https://www.ncbi.nlm.nih.gov/bioproject/ PRJNA1033378).
