# Supplementary material for: Genomic evidence for demographic fluctuations, genetic burdens and adaptive divergence in fourfinger threadfin Eleutheronema rhadinum
Source: Mar Life Sci Technol. 2025 Jan 27;7(1):66–78. doi: 10.1007/s42995-024-00276-4 (PMC11871173; doi:10.1007/s42995-024-00276-4)
Supplement: Supplementary file 1 — Supplementary file1 (PDF 521 KB) [file 42995_2024_276_MOESM1_ESM.pdf]

Genomic evidence for demographic fluctuations, genetic burdens and adaptive divergence in fourfinger threadfin *Eleutheronema rhadinum*

Jie Xiao[1,2] and Wen-Xiong Wang[1,2*]

[1]*School of Energy and Environment and State Key Laboratory of Marine Pollution, City University of Hong Kong, Kowloon, Hong Kong, China*

[2]*Research Centre for the Oceans and Human Health, City University of Hong Kong Shenzhen Research Institute, Shenzhen 518057, China*

*Corresponding author, Email: wx.wang@cityu.edu.hk

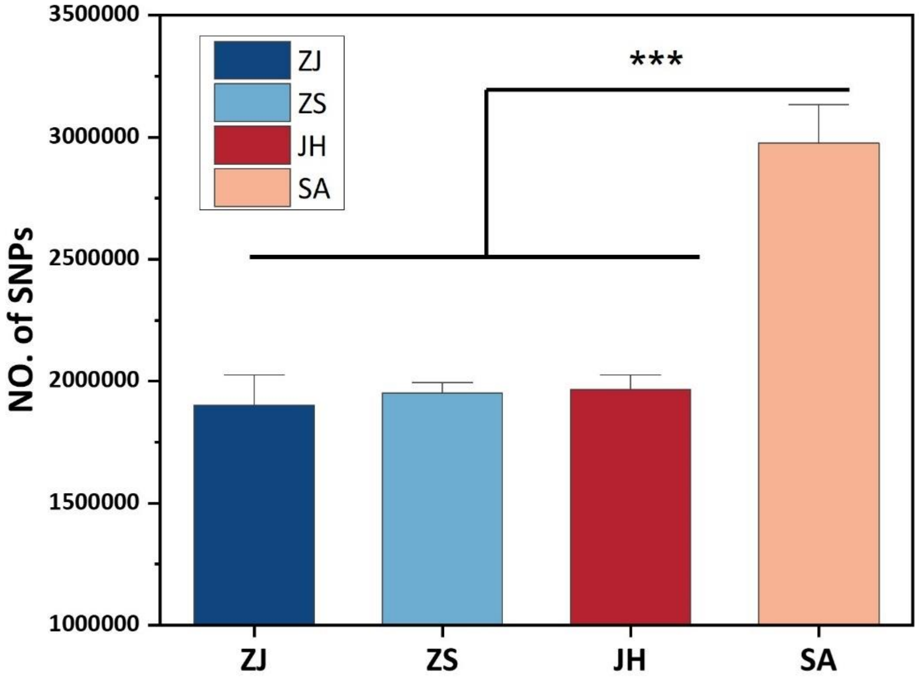

Figure S1. Number of SNPs in 72 wild *E. rhadinum* populations. Statistical significance of differences in the number of variants among species was conducted by the Mann-Whitney test.

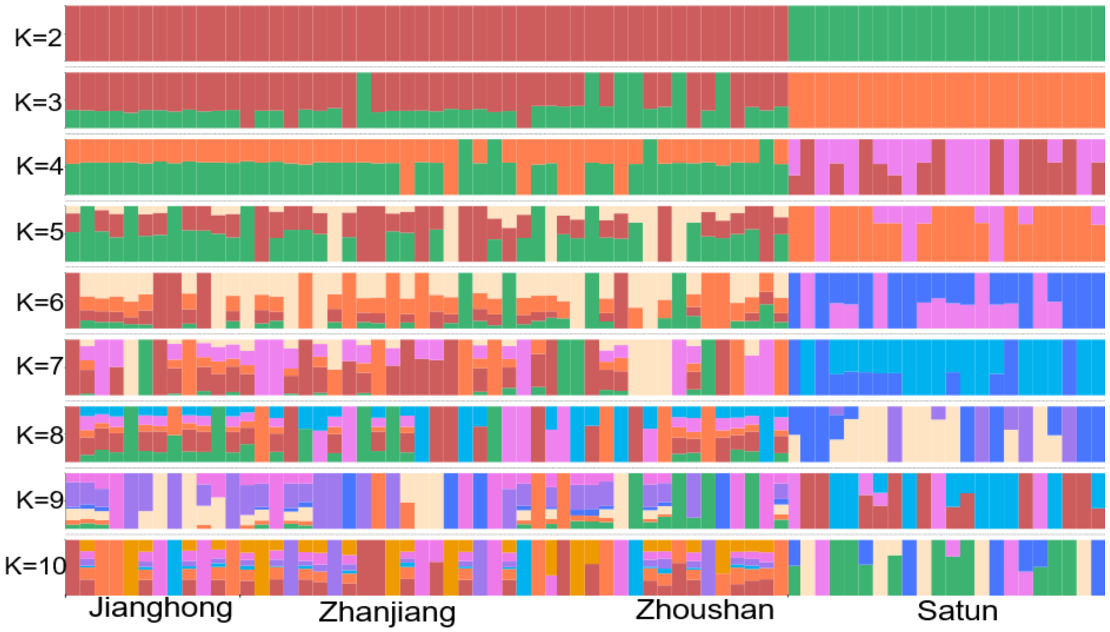

Figure S2. The length of each colored segment represents the proportion of the individual's genome from K = 2 to 10 ancestral populations. The geographic locations are at the bottom of the figure.

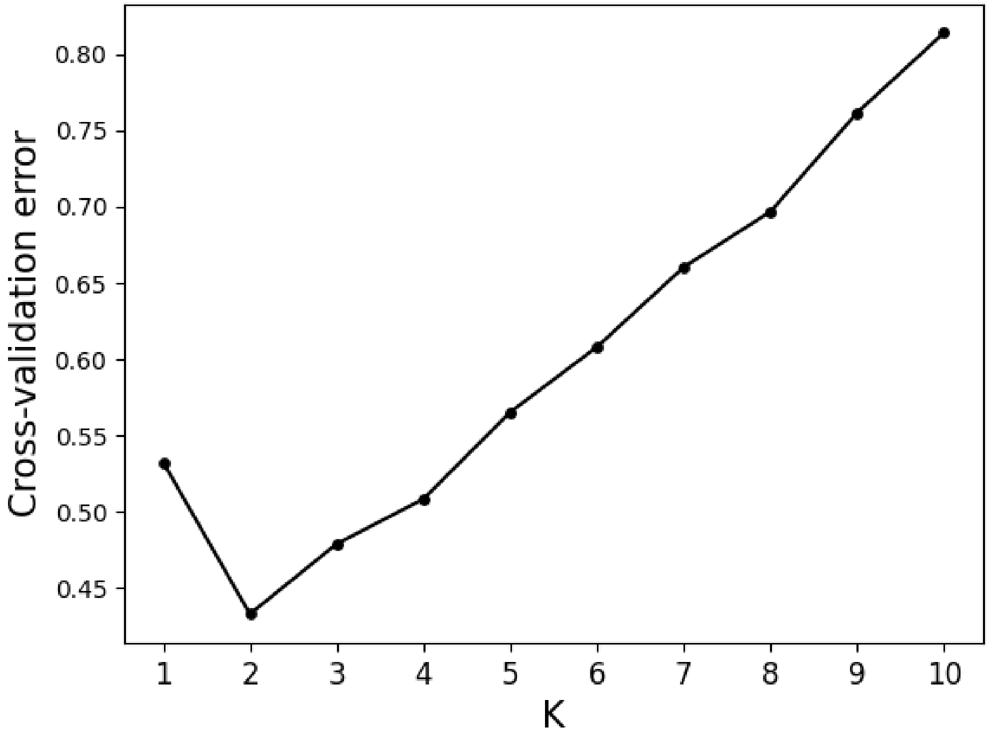

Figure S3. The cross-validation error (CV) of wild *E. rhadinum* populations used to explore the best K in the ADMIXTURE program with default parameters.

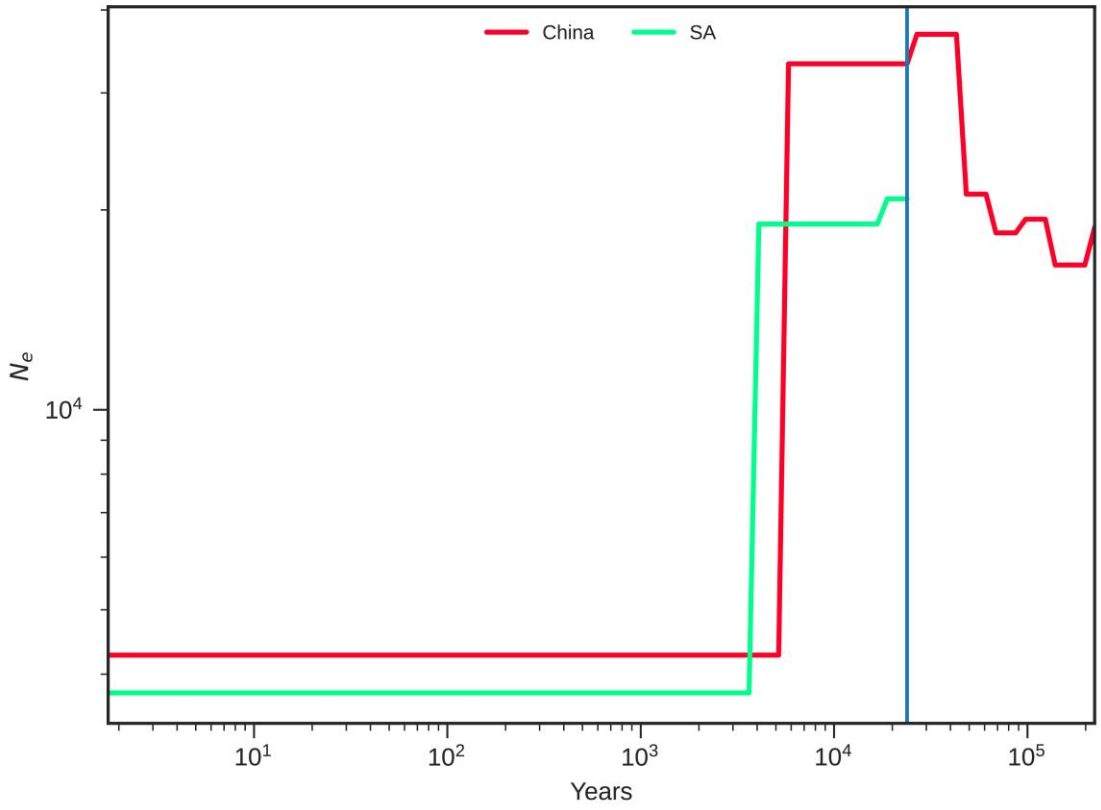

Figure S4. SMC++ inferences with 20 replicated runs for three China populations and SA of wild *E. rhadinum*. The dashed vertical line indicates the time of divergence (nearly 23, 000 yr ago).

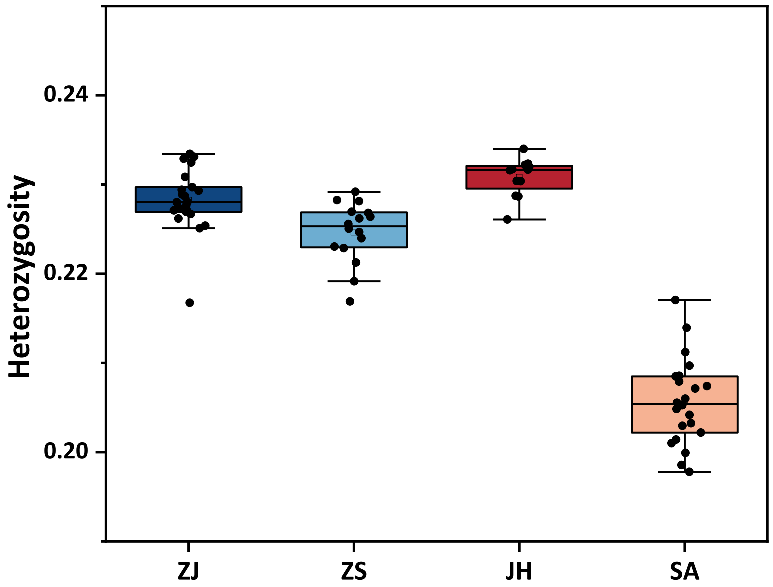

Figure S5. Individual heterozygosity was estimated for nonoverlapping 1Mb regions along the genome.

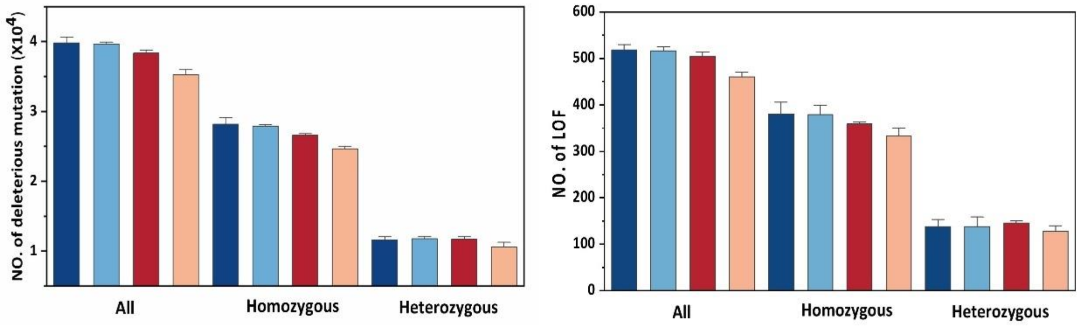

Figure S6. The derived mutations in the coding region include Loss of Function (LoF) and deleterious missense mutations.

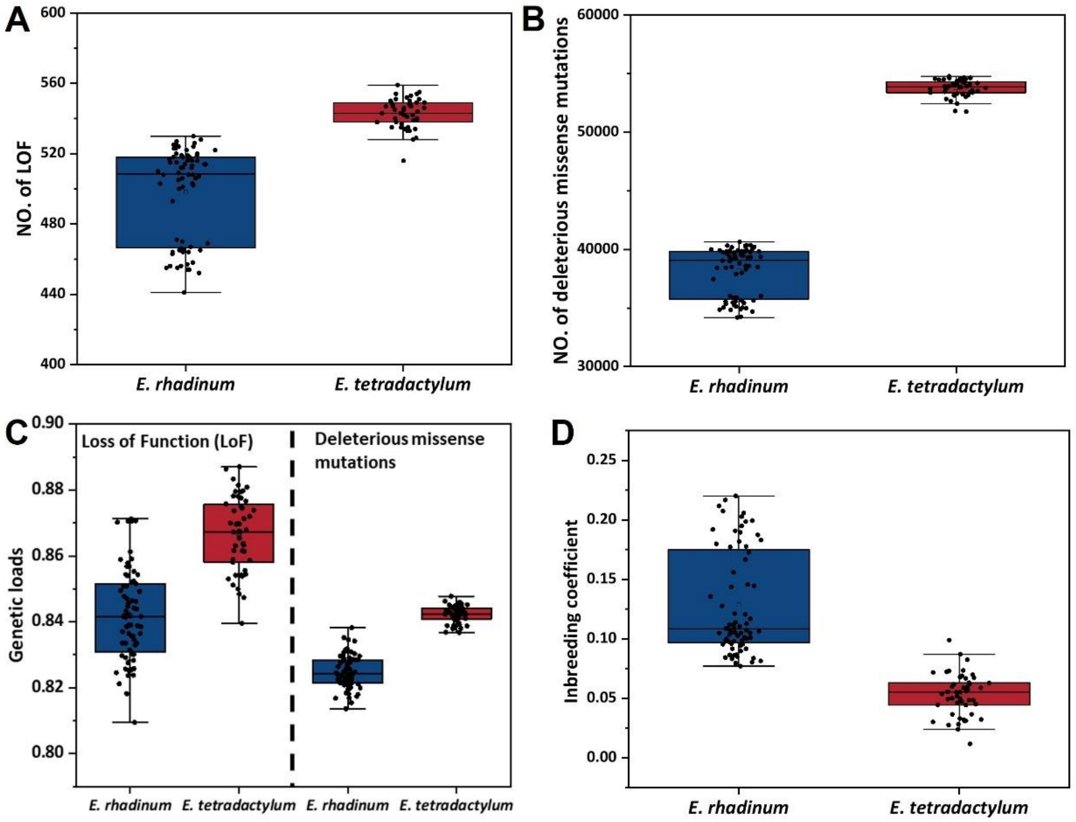

Figure S7. Comparasion of deleterious mutations and genetic load in *E. rhadinum* with that of its closely related sister species *E. tetradactylum*.

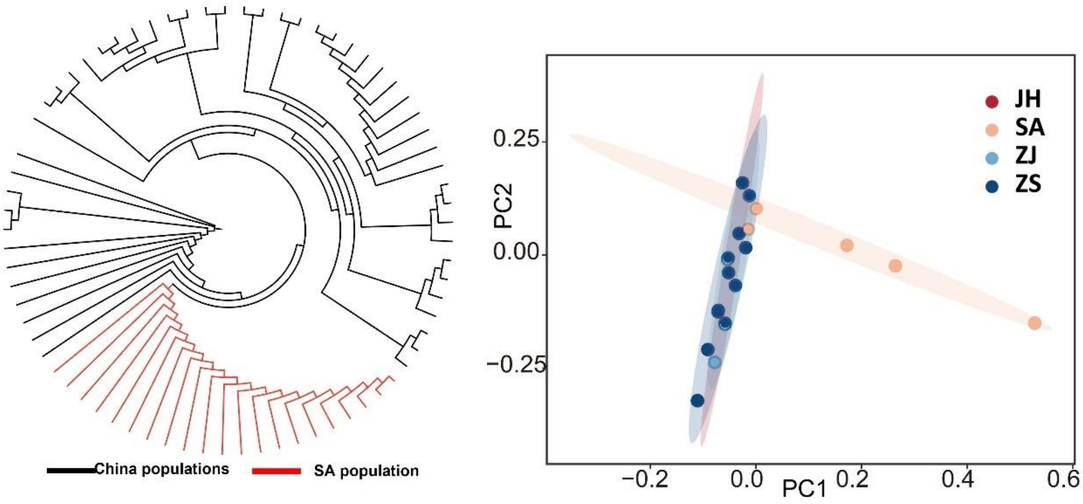

Figure S8. Characterization of one of the genes (*TRPM3*) in the highly diverged region. Phylogenetic tree (Left) and PCA (Right) of *TRPM3* among E. rhadinum populations.
